# Rapid Response in a Patient with Relapsed/Refractory Multiple Myeloma Treated with BRAF/MEK Inhibitors

**DOI:** 10.1155/2020/8821415

**Published:** 2020-12-11

**Authors:** Steve Biko Otieno, Syed Nasir, Alva Weir, Robert Johnson

**Affiliations:** ^1^The University of Tennessee Health Science Center, Department of Hematology/Oncology, 19 S. Manassas, Memphis, TN 38103, USA; ^2^The Veterans Affairs Medical Center, 1030 Jefferson Ave, Memphis, TN 38104, USA; ^3^The West Cancer and Research Institute, 7945 Wolf River Blvd, Germantown, TN 38138, USA

## Abstract

Patients with relapsed and refractory multiple myeloma have a poor prognosis. The mitogen-activated protein kinase (MAPK) pathway has been implicated in the pathogenesis of multiple myeloma. Several mutations in this pathway can lead to its constitutive activation leading to oncogenesis. One such mutation is BRAFV600E which is a therapeutic target in the treatment of melanoma, lung cancer, colon cancer, thyroid cancer, and hairy cell leukemia. BRAFV600E-directed therapy currently does not have approval in multiple myeloma. It has been proposed that this mutation leads to proteasome inhibitor resistance. About 4–10% of multiple myeloma cases harbor the BRAFV600E mutation. Herein, we report a case of a patient with relapsed and refractory multiple myeloma who had a progression-free survival (PFS) of 8.5 months on BRAF-targeted therapy.

## 1. Background

Multiple myeloma is a malignancy caused by neoplastic proliferation of atypical plasma cells that can cause hypercalcemia, renal failure, bone lesions, and anemia [1]. It is generally considered incurable with patients eventually requiring salvage therapy. Several drug classes have been shown to be effective in myeloma treatment and include proteasome inhibitors, immunomodulatory imide drugs (IMiDs), CD38 inhibitors, alkylating agents, histone deacetylase inhibitors, nuclear protein export inhibitors, anthracyclines, and steroids. Patients can also undergo autologous stem cell transplant. All these treatment options have improved overall survival in multiple myeloma [2]. However, some patients still develop relapsed and refractory disease despite these lines of treatment or are not good candidates for some of these therapies due to toxicities or comorbidities. There is therefore a need to expand the therapeutic armamentarium against multiple myeloma in the relapsed and refractory setting. Molecular analysis of multiple myeloma has revealed that about 4–10% of myeloma cases harbor BRAF mutations [3]. BRAF-directed therapy is used in melanoma, thyroid cancer, colon cancer, and hairy cell leukemia. While there are no approved BRAF-directed therapies in multiple myeloma, a few cases of off-label use have been reported [4–6].

We report a case of a patient with relapsed and refractory myeloma harboring the BRAFV600E mutation who was treated off-label with combination of BRAF and MEK inhibition with a gratifying response.

## 2. Case Report

The patient is a 68-year-old male with a past medical history notable for prostate cancer, in remission, who initially presented with high fevers, cough, and congestion. A computed tomography (CT) scan of his chest, abdomen, and pelvis showed a 4 centimeter (cm) lytic lesion within the manubrium and lytic lesions in the thoracic spine with the largest being a 1.4 cm T6 lesion and a 1.1 cm lucent T4 lesion. Also noted was a 1.1 cm lucent right iliac lesion. A subsequent positron emission tomography (PET) scan done a week later showed diffuse hypermetabolic lesions throughout the axial skeleton with prominent lesions in the manubrium, right ilium, cervical, thoracic and lumbar spines, sacrum, bilateral scapulae, and humeri. Multiple myeloma was suspected.

A protein electrophoresis revealed two M-bands 0.13 grams per deciliter (g/dL) and 3.37 g/dL (normal reference 0). Immunofixation showed IgG lambda light chain restriction. Kappa-free light chains were 1.9 milligrams per deciliter (mg/dL), and lambda-free chains were 124 mg/dL with a kappa to lambda ratio of 0.02 (reference range 0.26–1.65). Evaluation of immunoglobulins showed IgG of 7641 mg/dL (reference range 700–1600), IgA of 71 mg/dL (reference range 70–400), and IgM of 55 mg/dL (reference range 40–230). 24-hour urine protein was 5 grams with M protein comprising 90% of total urine protein. Calcium was 9 mg/dL (reference range 8.4–10.2). Antinuclear antibody (ANA), rheumatoid factor (RF), human immunodeficiency virus (HIV), and hepatitis B and C were all negative.

The lytic sternal lesion was biopsied, and a bone marrow biopsy was also done. The bone marrow biopsy revealed a 90–100% hypercellular marrow with IgG lambda-restricted plasma cells comprising 80% of the bone marrow cellularity. Cytogenetics showed a complex karyotype. Fluorescence *in situ* hybridization (FISH) showed del13q, 12p, 12q, 16q, and 22q and gain of 1q, 5, 6p, 7, 9, 15q, and Xq. Next generation sequencing showed BRAFV600E mutation with a 12% variant frequency and increased stainable iron. Congo red stain was negative. He was diagnosed with ISS stage III, high-risk multiple myeloma.

He was initially treated with lenalidomide-bortezomib-dexamethasone (RVd) regimen but was primary refractory with disease progression noted within 1 month. His case was discussed in the tumor board, and it was recommended the he should be started on a combination of dexamethasone, cisplatin, doxorubicin, cyclophosphamide, and etoposide (D-PACE). However, the patient declined this therapy. He was instead treated with pomalidomide, daratumumab, and dexamethasone. He had primary progression and was switched to pomalidomide, carfilzomib, and dexamethasone and had a response for 8 months before progression. His case was again reviewed, and it was decided to try a BRAF/MEK inhibitor combination. He started treatment with cobimetinib 60 mg daily on days 1–21 of a 28-day cycle and vemurafenib 960 mg twice a day for 28 days in a 28-day cycle. The patient was seen in clinic on weeks 5, 9, 13, 16, 25, and 37 after initiation of treatment. Progression was noted in week 37. His therapy was changed to selinexor.

**Figure 1:**
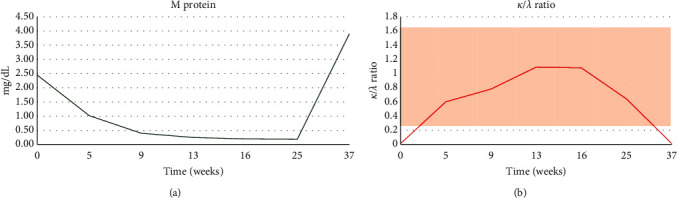
Response to treatment. (a) M protein levels after initiation of treatment with vemurafenib and cobimetinib at 0, 5, 9, 13, 16, 25, and 37 weeks. (b) Changes in kappa/lambda ratio at 0, 5, 9, 13, 16, 25, and 37 weeks. The beige box shows the normal range of the kappa/lambda ratio.

He tolerated therapy well. His only complaint was mild fatigue and occasional nausea controlled with antiemetics. His M protein levels and kappa/lambda ratio were as shown in Figure 1. His kappa/lambda light chain ratio normalized in 5 weeks. He had a PFS of 8.5 months.

## 3. Discussion

Multiple myeloma is generally considered incurable with most patients eventually requiring multiple lines of salvage therapy. Some patients can have limited treatment options due to toxicity or comorbidities. There is therefore a need for new treatment options.

The BRAF protein is part of the MAPK pathway, and BRAF mutations lead to constitutive activation of this pathway. BRAF mutation-directed therapies are used in solid malignancies including melanoma, colon cancer, and thyroid cancer. In B-cell malignancies, this mutation is found mainly in hairy cell leukemia where it is targeted for therapy in the relapsed refractory setting [7]. However, this mutation is rare in hematologic malignancies in general [8, 9]. About 4–10% of multiple myeloma cases harbor the BRAFV600E mutation [3]. While there are no completed large randomized clinical trials of BRAF inhibitors in multiple myeloma, a few cases of off-label use of BRAF inhibitors have been reported [4–6].

The patient in this case report progressed despite multiple lines of treatment. With a few cases of response to BRAF-directed therapy reported in multiple myeloma patients harboring BRAFV600E mutations, off-label treatment was deemed to be rational and reasonable for this patient. Studies in melanoma have demonstrated that targeting BRAF using a single-agent BRAF inhibitor eventually leads to acquired resistance through a variety of pathways, one of them being activation of the downstream MEK protein [10]. Combination therapy with BRAF inhibitors and MEK inhibitors has been shown to have better outcomes [10]. Extrapolating from these data in melanoma studies, we decided to treat with a combination of the BRAF inhibitor vemurafenib and the MEK inhibitor cobimetinib.

Since the optimal dose in myeloma is still unknown, we decided to treat at doses used in melanoma. The patient achieved a rapid response with the light chain ratio normalizing in 5 weeks and with the patient achieving a partial response in 5 weeks with a PFS of 8.5 months. Our case adds to the list of case reports that suggest that treatment of multiple myeloma harboring BRAFV600E mutations with BRAF/MEK inhibitors is a reasonable treatment option.

BRAF mutations are usually a poor prognostic indicator in other malignancies [11, 12]. In multiple myeloma, the prognostic significance is unclear. However, the study by Andrulis et al. reported a clinically more aggressive phenotype characterized with decreased OS in patients with BRAFV600E, and the study by Rustan et al. did not show any relation to a poor prognosis when compared to BRAF wild type. These differences may be due to different patient populations and small sample sizes [4, 13].

Shirazi et al. linked activating RAS and RAF mutations to enhanced proteasome assembly and capacity, thereby conferring proteasome inhibitor resistance in established myeloma cell lines. [14] This resistance was overcome by combination of BRAF and MEK inhibitors [14]. The initial bone marrow for our patient showed a BRAFV600E mutation with a variant frequency of 12% suggesting that it was a significant subclone but likely not the dominant clone. It is reasonable to surmise that the initial therapies conferred a growth advantage to BRAFV600E clone. Unfortunately, we do not have another biopsy to confirm if this clone had expanded prior to the BRAF inhibitor therapy.

Two clinical trials to assess the effectiveness of BRAF/MEK inhibition in relapsed/refractory multiple myeloma, NCT02834364 and NCT03091257, are currently recruiting. The results of these trials should provide further insight into the targeting of the MAPK pathway in treatment of multiple myeloma.

## 4. Conclusion

This case report suggests that targeting of the MAPK pathway through BRAF/MEK inhibition may be an additional therapeutic option in relapsed/refractory multiple myeloma.

## Data Availability

All data analyzed during this study are included in this published article.
